# Multiscale Method for Oseen Problem in Porous Media with Non-periodic Grain Patterns

**DOI:** 10.1007/s11242-016-0762-3

**Published:** 2016-09-06

**Authors:** Bagus Putra Muljadi

**Affiliations:** Department of Earth Science and Engineering, Imperial College, London, SW7 2BP UK

**Keywords:** Crouzeix–Raviart element, Oseen approximation, Multiscale finite element method, Penalisation method

## Abstract

Accurate prediction of the macroscopic flow parameters needed to describe flow in porous media relies on a good knowledge of flow field distribution at a much smaller scale—in the pore spaces. The extent of the inertial effect in the pore spaces cannot be underestimated yet is often ignored in large-scale simulations of fluid flow. We present a multiscale method for solving Oseen’s approximation of incompressible flow in the pore spaces amid non-periodic grain patterns. The method is based on the multiscale finite element method [MsFEM Hou and Wu in J Comput Phys 134:169–189, 1997)] and is built in the vein of Crouzeix and Raviart elements (Crouzeix and Raviart in Math Model Numer Anal 7:33–75, 1973). Simulations of inertial flow in highly non-periodic settings are conducted and presented. Convergence studies in terms of numerical errors relative to the reference solution are given to demonstrate the accuracy of our method. The weakly enforced continuity across coarse element edges is shown to maintain accurate solutions in the vicinity of the grains without the need for any oversampling methods. The penalisation method is employed to allow a complicated grain pattern to be modelled using a simple Cartesian mesh. This work is a stepping stone towards solving the more complicated Navier–Stokes equations with a nonlinear inertial term.

## Introduction

Modelling of flow through porous bodies is a topic of high importance in various fields of engineering, chemical, biological or geological applications. One of the most significant challenges persisting in virtually all these areas is the disparity between the spatial scales at which flow and transport can be understood; and the scales at which practical model predictions are needed (Scheibe et al. 2015). This disparity in scales forces a trade-off between building models which suffice for practical application, and models that solve the problem *ab-initio* but which may not be able to cope with large-scale problems adequately. To place this in context, in geological media, X-ray techniques now allow three-dimensional images to be acquired routinely (Blunt et al. 2013). The pore spaces of these rocks are typically of order microns across. However, for practical applications in oil recovery, carbon dioxide storage and contaminant transport, flow over 100s m to km needs to be predicted. This enormous range of scales precludes the use of a direct method that resolves pore-scale flow while determining reservoir-scale behaviour. Instead, techniques that can approximate the flow over distances much larger than the pore scale are needed. A number of multiscale simulation paradigms have been developed to bridge first-principles and empirical methods, and provide a link between micro, and macroscale models. An additional problem is that in many applications, such as flow in fractured rock and near-well bore flows, the nonlinear, or inertial term in the Navier–Stokes equation are significant. This means that at the large-scale, the application of the linear Darcy-law for flow is inaccurate.

Our choice of a particular multiscale method is based on the following. First, we consider direct Navier–Stokes simulation on the pore geometry as the holy-grail of microscale simulation for it is considered to be the most complex, and highly resolved spatially (although Navier–Stokes itself can be seen as an upscaled representation of molecular-scale interactions, with effective parameters such as viscosity and density). Such a microscale model strikes a balance between the appropriate level of complexity with current technological advances. For example, recent developments in both computational algorithms, and increases in computer power, coupled with the availability of pore-scale images, have enabled the routine prediction of permeability, with direct Navier–Stokes calculations on samples containing up to a billion voxels (Blunt et al. 2013; Mostaghimi et al. 2012). Second, we are interested in a method capable of resolving the microscale model directly over the domain of interest without losing any degrees of freedom—which rules out other multiscale methods which borrow their philosophy from homogenisation theory (e.g formal upscaling with closure approximation). Several multiresolution solvers are designed for this purpose, i.e to provide computationally efficient ways of obtaining a complete solution on the fine grid, for example: multigrid solvers and preconditioners (Wesseling 1992), multiscale finite element methods (MsFEM; Hou and Wu 1997; Aarnes et al. 2005; Jenny et al. 2003), and multiscale mimetic methods (Lipnikov et al. 2011). We choose to develop an adaptation of MsFEM dedicated for solving flow in a pore domain left void by non-periodic grain patterns, which is a representation of all natural pore structures.

The challenge in applying MsFEM in a non-periodic setting is to avoid an intersection between a coarse element boundary and a grain. On the other hand, the overall performance of MsFEM rely on the accuracy of the multiscale basis function which is very sensitive the treatment of subgrid boundary condition. The application of oversampling methods (Efendiev et al. 2013; Chu et al. 2008; Henning and Peterseim 2013) was intended to circumvent this problem by broadening the domain in which basis functions are sampled. While the methods perform satisfactorily in the context of perforated media (Bris et al. 2014; Chung et al. 2015), nevertheless it necessitates an *ad hoc* parameterisation and results in a larger computational problem. Another alternative is to adopt a nonconforming finite element method and impose only a *weak* continuity between coarse element boundaries and therefore allowing the coarse element boundaries to adapt to random patterns of grains. In our previous works, the nonconforming Crouzeix–Raviart element has been adopted successfully for solving advection–diffusion and Stokes equations (Bris et al. 2013; Degond et al. 2015; Muljadi et al. 2015).

Creeping or Stokes flow is often assumed in porous media. This ceases to apply, as mentioned above, for example, near propped fractures, or boreholes in reservoirs where inertial forces becomes dominant. Even in the absence of fractures, Muljadi et al. (2016b) studied the non-Darcy flow behaviour in porous media with different pore heterogeneities and found that the cessation of the Darcy relationship in Estaillades limestone already takes place at Re $\approx 0.001$, three orders of magnitude smaller than what suggested in the literature (Re $\approx 1$) based on studies of homogeneous media, such as bead packs, which are poor representations of the heterogeneous reservoir rocks of practical interest.

The flow of viscous fluid is adequately governed by Navier–Stokes equation

1 $$\begin{aligned} \mu \nabla ^2\mathbf {u}+\rho (\mathbf {u}\cdot \nabla \mathbf {u})-\nabla p= & {} \mathbf {f},\nonumber \\ \nabla \cdot \mathbf {u}= & {} 0, \end{aligned}$$

where $\mathbf {u}$ and *p* are velocity and pressure, respectively, $\mu$ is the dynamic viscosity, and $\rho$ is the density. Typically the nonlinear, inertial term $\rho (\mathbf {u}\cdot \nabla \mathbf {u})$ would lead to nonlinear algebraic equations which require iterations of the linearised equation to solve. Our ultimate goal is to study the application of MsFEM on solving (1) in both fine and coarse-scales. However, we find it reasonable to first apply our method on the Oseen’s approximation of Navier–Stokes equation where the inertial term is substituted by Oseen term $\rho (\mathbf {U}\cdot \nabla \mathbf {u})$, where $\mathbf {U}$ is a known velocity vector. Proudman and Pearson (1957) point out that Oseen’s solution provides a uniformly valid *zeroth* order approximation to the Navier–Stokes equation at finite Reynolds number. Oseen system includes important inertial factors, while retaining the solution linearity, and thus simplicity (Oseen equation is analytically tractable). As a result, Oseen system has been utilised as a *testbed* for validating different computational approaches (e.g. stabilisation of finite element discretisations (Abraham et al. 2004), weak Galerkin finite element method (Liu et al. 2016), or artificial boundary conditions on truncated computational domains (Chun-xiong 2002) for it represents an alternative model to the Stokes’ in which the nonlinear term is eliminated entirely.

In the context of multiscale methods for flow in porous media, Abdulle and Bud (2015) presented a multiscale method based on the coupling of an effective Darcy equation on a macroscopic mesh with unknown permeabilities recovered from micro finite element calculations for Stokes problems. Alyaev et al. (2014) presented a heterogeneous multiscale method that utilises fine-scale information directly to solve problems for general single-phase flow on the Darcy scale. Their work focuses on the nonlinear flow regime, but does not employ Oseen’s approximation. Bonfigli and Jenny (2009) presented a multi-scale pressure solver for the incompressible Navier–Stokes equations with immersed boundaries. For two-phase flows in porous media, Tomin and Lunati (2013) presented hybrid Multiscale Finite Volume (MsFV) method, which couples pore-scale and Darcy descriptions of two-phase flow in porous media. The finite volume discretisation ensures conservation of mass at the coarse-scale, and a conservative pore-scale velocity field. The above-mentioned methods apply *Darcy modelling* at the coarse-scale whereas in this paper, Oseen equations are solved at both fine, and coarse-scales.

To avoid having to work with complicated boundary-fitted or even unstructured meshes, we employ the penalisation method (Angot et al. 1999) when modelling non-periodic grain patterns. Here, we simply *force* the solution to vanish within the grain boundaries. Consequentially, this approach allows the modelling of a complicated grains pattern on a simple Cartesian mesh.

This paper is organised as follows. The formulation of the problem is given in Sect. 2. The construction of Crouzeix–Raviart elements is presented in Sect. 3. In Sect. 4, the application of penalisation method on our problem is described. Then, the description of the computation of the reference solutions is given in Sect. 5 followed by some remarks on the treatment of the boundary condition in Sect. 6. Numerical tests are presented and the results discussed in Sect. 7 followed by some concluding remarks.

**Fig. 1:**
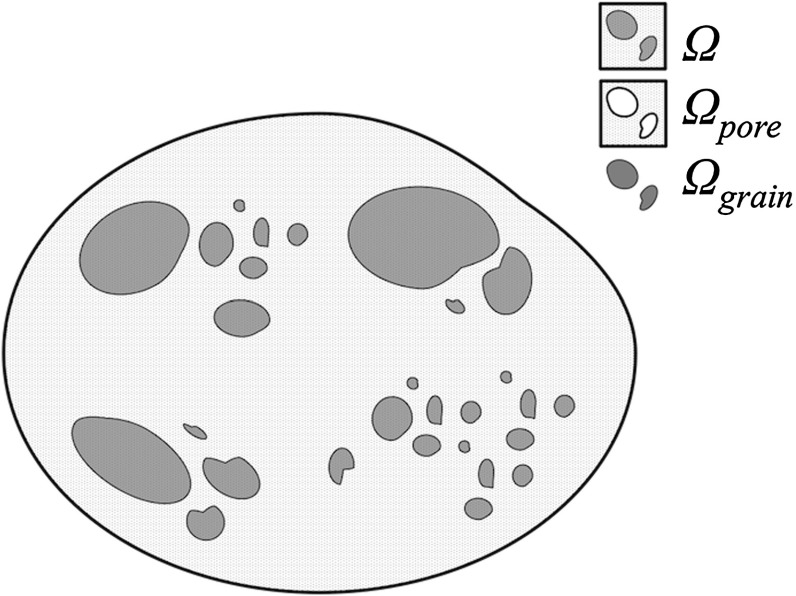
An illustration of a domain $\varOmega$ consisting of a pore domain $\varOmega _\text {pore}$, and a grain domain $\varOmega _\text {grain}$

## Problem Formulation

We define $\varOmega$, a two-dimensional domain consisting of a grain domain $\varOmega _\text {grain}$, and a pore domain $\varOmega _\text {pore}$ perforated by grains, see Fig. 1. Then, let $\varepsilon$ denote the diameter of the smallest grain. The steady-state Oseen’s problem is to find velocity $\mathbf {u}$ which is the solution to:

2 $$\begin{aligned} \mu \nabla ^2\mathbf {u}+\rho (\mathbf {U}\cdot \nabla \mathbf {u})-\nabla p= & {} \mathbf {f} \quad \text {in}\quad \varOmega _{\text {pore}}\nonumber \\ \nabla \cdot \mathbf {u}= & {} 0 \quad \text {in} \quad \varOmega _{\text {pore}}. \end{aligned}$$

The boundary condition of Eq. (2) is given by:

3 $$\begin{aligned} \mathbf {u}= & {} \mathbf {g} \text {, on } \partial \varOmega \cap \partial \varOmega _{\text {pore}}, \nonumber \\ \mathbf {u}= & {} \mathbf {U} \text {, when } \{x,y\}\rightarrow \infty , \end{aligned}$$

where $\mathbf {f}$ is a source function, and $\mathbf {g}$ is a function fixed at the boundary $\partial \varOmega$. In this paper, we consider only a no-slip condition on the grain boundaries: $\mathbf {u}=0, \text { on }{\partial \varOmega _{\text {grain}}}$.

**Fig. 2:**
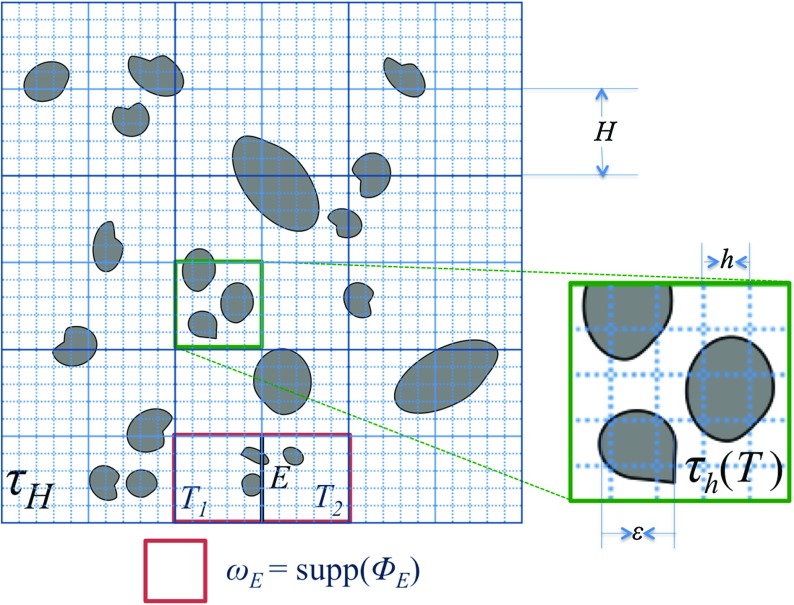
An illustration of the discretised domain $\tau _H$, and $\omega _{E}$ which is the support space for $\mathbf {\Phi }_{E}$. In all applications in this paper, $\varepsilon /h\ge 5$ is maintained

## Application of Crouzeix–Raviart MsFE

Here, we explain the application of our method starting from the definition of the coarse and fine meshes. We then introduce the functional spaces for our multiscale basis functions and describe the construction of these functions within each coarse elements.

### Discretisation

We discretise $\varOmega$ into a two-dimensional homogeneous Cartesian coarse mesh $\tau _H$ (see Fig. 2). $\tau _H$ consists of coarse elements $T_k, k=1,2,\dots ,{N_H}$, where $N_H$ is the total number of coarse elements, each with width *H*. We define $\mathcal {E}_H$ the set of all coarse edges $E_j,j=1,2,\dots ,{N_E}$ in $\tau _H$ including the edges on the domain boundary $\partial \varOmega$. For each element *T*, we construct a fine mesh $\tau _h(T)$, consisting of fine elements each with width *h*. Note that the combination of $\tau _h(T)$ for all $T\in \tau _H$ constructs a global fine mesh $\tau _h$ which overlaps with $\tau _H$. Conversely, one can generate $\tau _H$ from $\tau _h$ since the difference between the two meshes is only in the indexing.

### Crouzeix–Raviart Functional Spaces

The functional spaces for velocity $V_H$, and for pressure $M_H$ are given below:

4 $$\begin{aligned} M_H= & {} \{{q}\in L^2 \, \text{ such } \text{ that } \, q = \text{ constant, } \forall T \in \tau _H \},\\ V_H= & {} \{\mathbf {v}:\varOmega \rightarrow R^2 \, : \, \forall T \in \tau _H \,\text{ such } \text{ that }\nonumber \\ \mu \nabla ^2 \mathbf {v} + \rho (\mathbf {U}\cdot \nabla \mathbf {v}) -\nabla s= & {} 0 \text {, in }\varOmega _\text {pore}\cap T \nonumber \\ \nabla \cdot \,\mathbf {v}= & {} \text { constant in }\varOmega _\text {pore}\cap T\nonumber \\ \nabla \mathbf {v}\,n-sn= & {} \text { constant on }E\cap \varOmega _\text {pore}, \ \forall E\in \mathcal {E}_H(T)\},\nonumber \end{aligned}$$

where $\mathcal {E}_H(T)$ is the ensemble of edges composing $\partial T$. The key here is to maintain the continuity of (only) the average of $\mathbf {v}$ across an edge *E*: $\int _E [[\mathbf {v}]]=0$, where [[*v*]] is the *jump* of the value *v* across *E*. We wish to retain the advantage of our approach which has been successfully applied on Advection-Diffusion, and Stokes problems, namely: the weak imposing of continuity across element boundaries allows adaptive boundary conditions which relaxes the sensitivity of our method to random arrangements of grains, without the need of applying the more cumbersome oversampling methods.

### Construction of a Crouzeix–Raviart Basis

For each edge $E\in \mathcal {E}_{H}$ we construct $\mathbf {\Phi }_{E,i}\in V_{H}$, such that $\int _{E}\mathbf {\Phi }_{E,i}=\mathbf {e}_{i}$, and $\int _{E^{\prime }}\mathbf {\Phi }_{E,i}=0$ for all $E^{\prime }\in \mathcal {E}_{H}$, $E^{\prime }\not =E$. These functions form a basis of $V_{H}$, i.e

5 $$\begin{aligned} V_{H}=\text {span}\{\mathbf {\Phi }_{E,i},~E\in \mathcal {E}_{H},~i=1,2\}. \end{aligned}$$

We also define $\text {supp}(\mathbf {\Phi }_{E,i})\subset \omega _{E}$, the ensemble of two quadrangles $T_k,k=1,2$ in $\tau _{H}$ which share an edge *E*. Hence find, in each of these quadrangles, $\mathbf {\Phi }_{E,i}$ and $\pi _{E,i}$, which are the solutions to:

6 $$\begin{aligned} \mu \nabla ^2 \mathbf {\Phi }_{E,i}+\rho (\mathbf {U}\cdot \nabla \mathbf {\Phi }_{E,i})-\nabla \pi _{E,i}= & {} 0,\text { in }\varOmega _\text {pore}\cap T_{k}, \\ \nabla \cdot \mathbf {\Phi }_{E,i}= & {} \text {constant, in }\varOmega _\text {pore}\cap T_{k}, \nonumber \\ \mathbf {\Phi }_{E,i}= & {} 0,\text { in }\varOmega _\text {grain}\cap T_{k}, \nonumber \\ \nabla \mathbf {\Phi }_{E,i}n-\pi _{E,i}n= & {} \text {constant, on }F\cap \varOmega _\text {pore}, \forall F\in \mathcal {E}_H(T_{k}), \nonumber \\ \int _{F}\mathbf {\Phi }_{E,i}= & {} \left\{ \begin{array}{c} \mathbf {e}_{i},~F=E \\ 0,~F\not =E\end{array}\right. ,\forall F\in \mathcal {E}_H(T_{k}),\nonumber \\ \int _{\varOmega _\text {pore}\cap T_{k}}\pi _{E,i}= & {} 0,\nonumber \end{aligned}$$

where $\mathcal {E}_H(T_{k})$ is the set of all the edges of the quadrangle $T_k$. To solve Eq. (6), we use Q1-Q1 finite element spaces in which both velocity and pressure degrees of freedom are defined on the same set of grid points. This arrangement is chosen due to the ease of programming and the computational efficiency. A stabilisation method is however necessary when this approach is considered. In a homogeneous Cartesian coordinate with fine element width of *h*, a stable solution can be achieved by perturbing the condition $\nabla \cdot \mathbf {\Phi }_{E,i} = 0$ with a pressure Laplacian term (see Brezzi and Fortin 1991). In a weak form, Eq. (6) reduces to finding $\mathbf {\Phi }_{E,i}\in H^{1}(T_{k}\cap \varOmega _\text {pore})$, $\pi _{E,i}\in L^2_{0}(T_{k}\cap \varOmega _\text {pore})$, and the Lagrange multipliers $\mathbf {\lambda }_{F}$, $\forall F\in \mathcal {E}_H(T_{k})$, satisfying

7 $$\begin{aligned}&\int _{T_k} \mu \nabla \mathbf {\Phi }_{E,i} :\nabla \mathbf {v}_h +\int _{T_k} \rho (\mathbf {U}\cdot \mathbf {\Phi }_{E,i})\cdot \mathbf {v}_h -\int _{T_k}\pi _{E,i}\nabla \cdot \mathbf {v}_h \nonumber \\&\qquad +\sum _{F\in \mathcal {E}_H(T_{k})}\mathbf {\lambda }_{F}\cdot \int _{F}\mathbf {\Phi }_{E,i} =0,\nonumber \\&\qquad -\int _\varOmega q_h\nabla \cdot \mathbf {\Phi }_{E,i}-\theta h^2 \int _\varOmega \nabla \pi _{E,i} \cdot \nabla q_h = 0,\nonumber \\&\qquad \sum _{F\in \mathcal {E}_H(T_{k})}\mathbf {\mu }_{F}\cdot \int _{F}\mathbf {\Phi }_{E,i} =\mathbf {\mu }_{E}\cdot \mathbf {e}_{i}, \quad \forall \mathbf {\mu }_{F}\in \text {R}^{2},~F\in \mathcal {E}_H(T_{k}). \end{aligned}$$

Here, $\mathbf {v}_h$ and $q_h$ occupy the same finite element spaces as $\mathbf {\Phi }_{E,i}$ and $\pi _{E,i}$, respectively. $\theta$ is the stabilisation parameter which we set as 0.01 for all our simulations (see Brezzi and Pitkäranta 1984).

### Crouzeix–Raviart MsFEM Coarse-Scale Solution

Here, we describe the coarse-scale solution of Eq. (2). By discretising *p* into $p_H$ and $\mathbf {u}$ into $\mathbf {u}_H$, we can solve Eq. (2) in $\tau _H$ and rewrite it in a weak form as:

8 $$\begin{aligned} a(\mathbf {u}_H,\mathbf {v}_H) + c(\mathbf {u}_H,\mathbf {v}_H) + b(\mathbf {v}_H,p_H)= & {} (\mathbf {v}_H,\mathbf {f}) ,\quad \forall \mathbf {v}_H \in V_H\nonumber \\ b(\mathbf {u}_H,q_H)= & {} \quad 0,\quad \forall q_H \in M_H \end{aligned}$$

where

9 $$\begin{aligned} a(\mathbf {u},\mathbf {v})= & {} \int _{\varOmega _\text {pore}} \mu \nabla \mathbf {u}: \nabla \mathbf {v}\, d{\varOmega _\text {pore}} \end{aligned}$$

10 $$\begin{aligned} c(\mathbf {u},\mathbf {v}) = c(\mathbf {U};\mathbf {u},\mathbf {v})= & {} \int _{\varOmega _\text {pore}}\rho (\mathbf {U}\cdot \nabla \mathbf {u})\cdot \mathbf {v}\, d{\varOmega _\text {pore}} \end{aligned}$$

11 $$\begin{aligned} b(\mathbf {v},q)= & {} -\int _{\varOmega _\text {pore}} q \nabla \cdot \mathbf {v}\, d{\varOmega _\text {pore}}. \end{aligned}$$

**Fig. 3:**
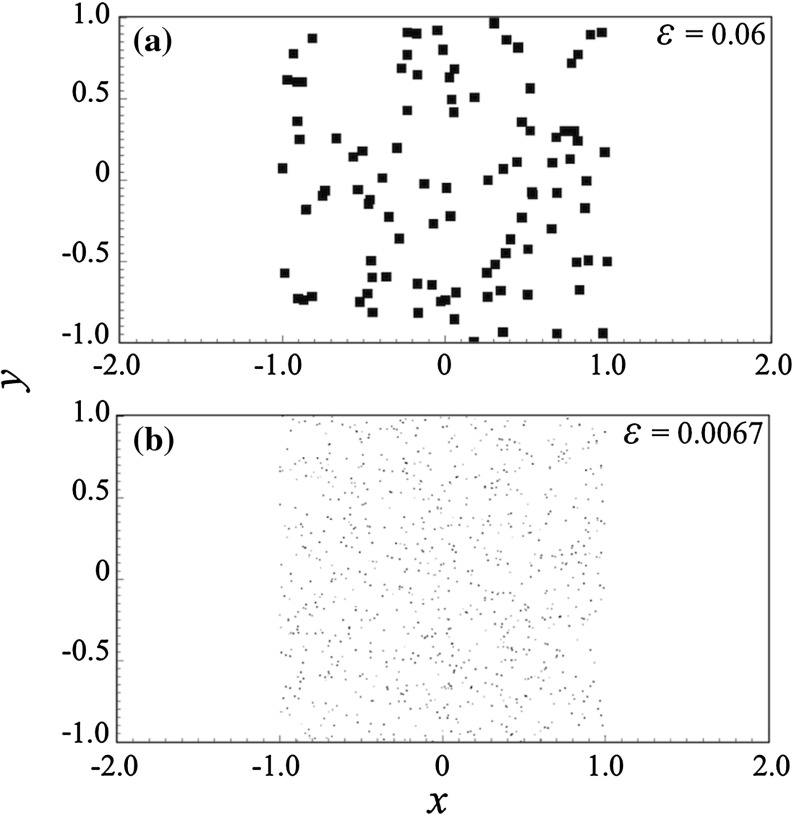
Two grain patterns in a channel consisting of randomly laid **a** 100 rectangular grains with width $\varepsilon = 0.06$; and **b** 900 rectangular grains with $\varepsilon = 0.0067$

The solution to problem (2) can then be approximated as linear combination of multiscale basis functions $\mathbf {\Phi }_{E,j}={\Phi }_{E}\mathbf {e}_j$, $j = 1,2$ with $\{\mathbf {e}_{1},\mathbf {e}_{2}\}$ being the canonical basis of $\mathcal {R}^{2}$, such that (Fig. 3):

12 $$\begin{aligned} \mathbf {u}_H(x,y)=\sum _{E\in \mathcal {E}_H,\,j=1,2}{u}_{E,j}\mathbf {\Phi }_{E,j}(x,y). \end{aligned}$$

## Penalisation Method

**Fig. 4:**
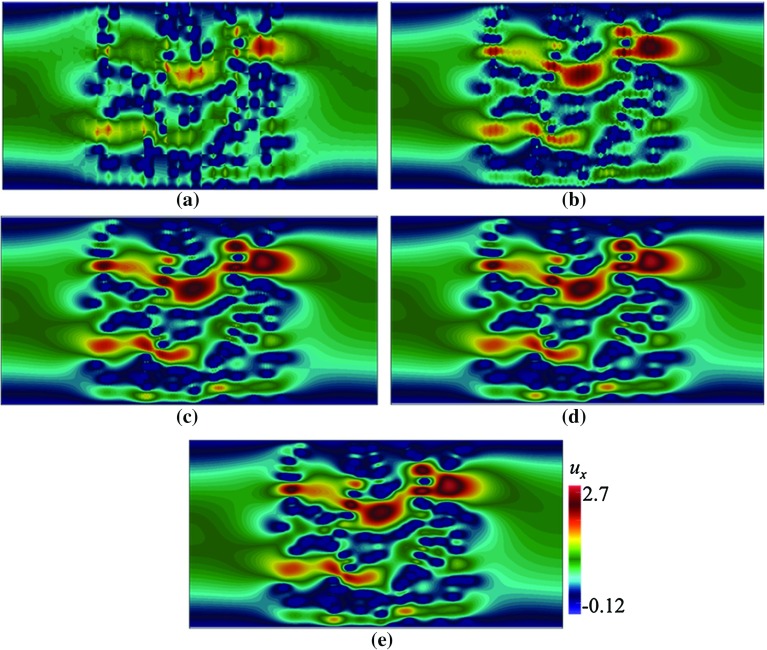
Contours of $u_x$ in a domain depicted in Fig. 3a, with $\mathbf {U}=(0.002,0)$ computed using Crouzeix–Raviart MsFEM on **a** $32\times 16$; **b** $64\times 32$; **c** $128\times 64$; **d** $256\times 128$ coarse elements; and **e** the reference solution

Often $\varOmega _\text {pore}$ is a complicated pore structure in which solving Eq. (2) may require a complicated boundary-fitted, or even an unstructured mesh. In order to confine our computations in a homogeneous Cartesian mesh, we employ the penalisation method (Angot et al. 1999). Henceforth, instead of solving Eq. (2) directly in $\varOmega _\text {pore}$, we solve (Fig. 4):

13 $$\begin{aligned} \mu _\kappa \nabla ^2 \mathbf {u}+\rho _\kappa (\mathbf {U}\cdot \nabla \mathbf {u}) + \sigma _\kappa \mathbf {u} - \nabla p= & {} \mathbf {f}_\kappa \text {, in } \varOmega \nonumber \\ \nabla \cdot \mathbf {u}= & {} 0 \text {, in }\varOmega \nonumber \\ \mathbf {u}= & {} \mathbf {g} \text {, on } \partial \varOmega \end{aligned}$$

in which

14 $$\begin{aligned} \mu _\kappa ;\rho _\kappa = \left\{ \begin{array}{cc} \frac{1}{h} &{} \text{ in } \varOmega _\text {grain} \\ \mu ;\rho &{} \text{ in } \varOmega _\text {pore} \end{array} \right. , \sigma _\kappa = \left\{ \begin{array}{cc} \frac{1}{h^3} &{} \text{ in } \varOmega _\text {grain} \\ 0 &{} \text{ in } \varOmega _\text {pore} \end{array} \right. , \mathbf {f}_\kappa = \left\{ \begin{array}{cc} 0 &{} \text{ in } \varOmega _\text {grain} \\ \mathbf {f} &{} \text{ in } \varOmega _\text {pore} \end{array} \right. . \end{aligned}$$

In our simulations, the chosen fine-scale element width *h* always satisfies $\varepsilon /h\ge 5$. The penalisation coefficient $\sigma _\kappa$ then forces the solution $\mathbf {u}$ to vanish inside the obstacles. Other variants of penalisation methods are studied in Angot et al. (1999).

## Reference Solution

**Fig. 5:**
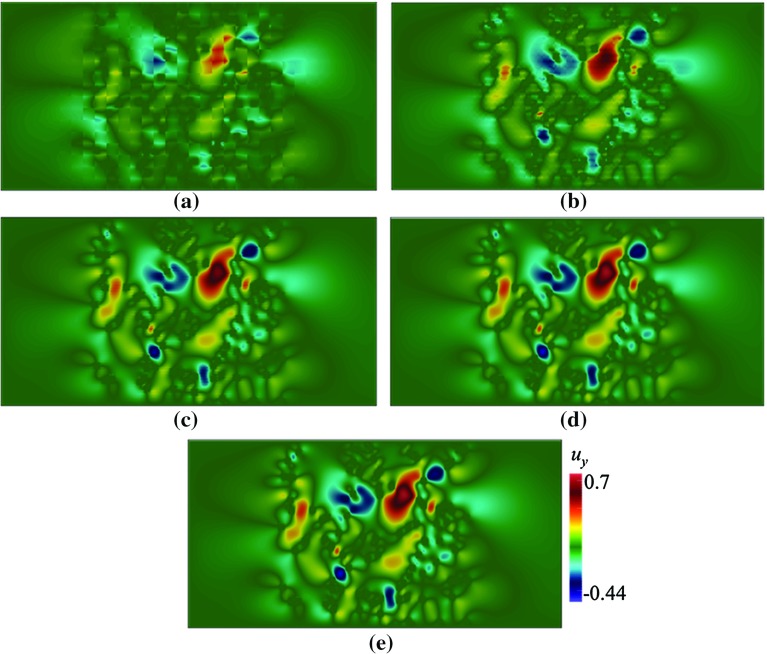
Contours of $u_y$ in a domain depicted in Fig. 3a, with $\mathbf {U}=(0.002,0)$ computed using Crouzeix–Raviart MsFEM on **a** $32\times 16$; **b** $64\times 32$; **c** $128\times 64$; **d** $256\times 128$ coarse elements; and **e** the reference solution

We use a Q1–Q1 finite element method to compute the reference solutions, as we do for computing $\mathbf {\Phi }$, in the global fine mesh $\tau _h$. In a weak form, the solution to Eq. (2) are $\mathbf {u}_h$ and $p_h$ such that (Fig. 5)

15 $$\begin{aligned} \int _\varOmega \mu _\kappa \nabla \mathbf {u}_h:\nabla \mathbf {v}_h +\int _\varOmega \rho _\kappa (\mathbf {U}\cdot \nabla \mathbf {u}_h)\cdot \mathbf {v}_h +\int _\varOmega \sigma _\kappa \mathbf {u}_h\cdot \mathbf {v}_h\nonumber \\ -\int _\varOmega p_h\nabla \cdot \mathbf {v}_h = \int _\varOmega \mathbf {v}_h \cdot \mathbf {f}_\kappa ,\nonumber \\ -\int _\varOmega q_h\nabla \cdot \mathbf {u}_h-\theta h^2(\nabla p_h,\nabla q_h) = 0, \end{aligned}$$

where $\mathbf {v}_h$, and $p_h$ occupy Q1-Q1 finite element spaces. We use the stabilisation parameter $\theta =0.01$ throughout this paper (Elman et al. 2005). Note that other stable or stabilised elements can be used to provide the reference solution.

## Boundary Condition

Note that the boundary condition $\mathbf {u}=\mathbf {g}$ on $\partial \varOmega$ is not included in Eq. (4). This is possible since we approximate the boundary condition in Eq. (6) only in a weak sense, i.e

16 $$\begin{aligned} \int _{E} \mathbf {u}_H = \int _{E} \mathbf {g}, \quad \forall E \in \mathcal {E}_H \quad \text {on}\quad \partial \varOmega . \end{aligned}$$

**Fig. 6:**
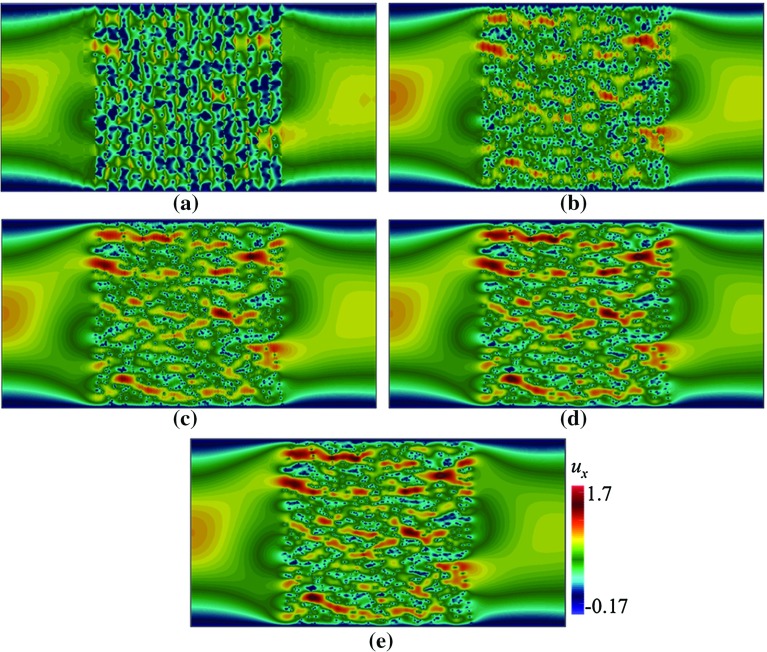
Contours of $u_x$ in a domain depicted in Fig. 3b, with $\mathbf {U}=(0.002,0)$ computed using Crouzeix–Raviart MsFEM on **a** $32\times 16$; **b** $64\times 32$; **c** $128\times 64$; **d** $256\times 128$ coarse elements; and **e** the reference solution

Together with Eq. (12), we apply on the boundary $\partial \varOmega$ (Fig. 6):

17 $$\begin{aligned} u_{E,i} = \int _{E} {\mathbf {g}\cdot \mathbf {e}_i}. \end{aligned}$$

This approach has been applied successfully in Muljadi et al. (2015) for the Stokes equation and is a modification with respect to previous work (Bris et al. 2013, 2014) where the boundary conditions were strongly incorporated in the definition of $V_H$. Our approach therefore gives more flexibility when implementing non zero $\mathbf {g}$, i.e every basis functions $\mathbf {\Phi }_{E,i}$ including those on boundary $\partial \varOmega$ can be computed in the same fashion—according to Eq. (6).

## Numerical Results

**Fig. 7:**
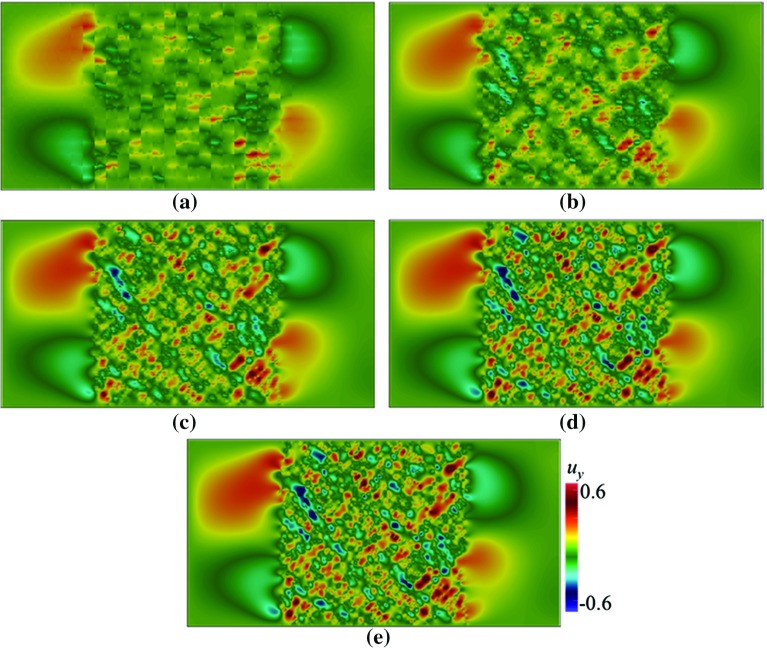
Contours of $u_y$ in a domain depicted in Fig. 3b, with $\mathbf {U}=(0.002,0)$ computed using Crouzeix–Raviart MsFEM on **a** $32\times 16$; **b** $64\times 32$; **c** $128\times 64$; **d** $256\times 128$ coarse elements; and **e** the reference solution

**Fig. 8:**
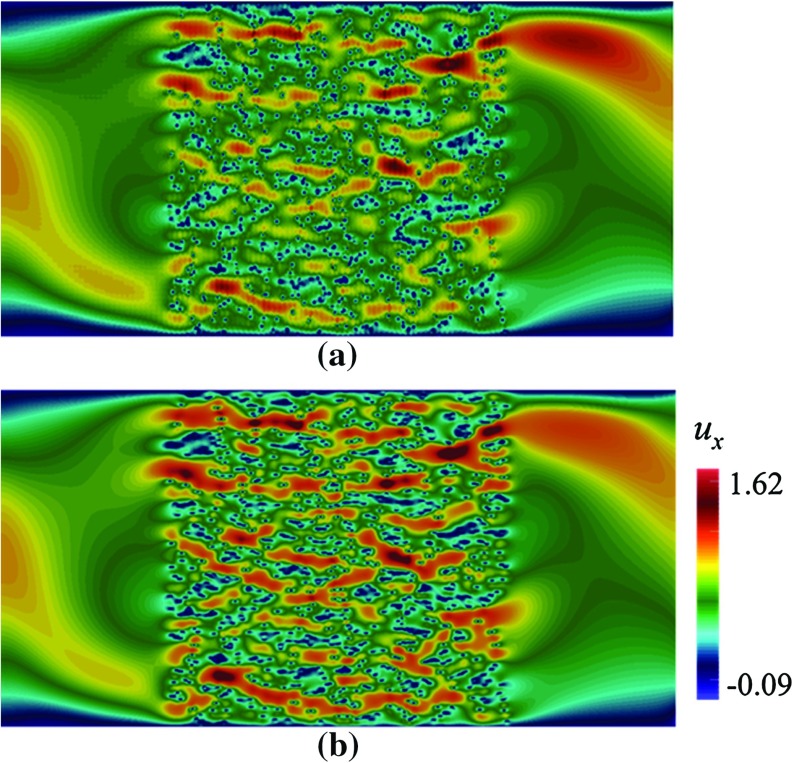
Contours of $u_x$ in a domain depicted in Fig. 3b, with $\mathbf {U}$ according to Eq. (18), computed using Crouzeix–Raviart MsFEM on **a** $128\times 64$ coarse elements; compared with **b** the reference solution

**Fig. 9:**
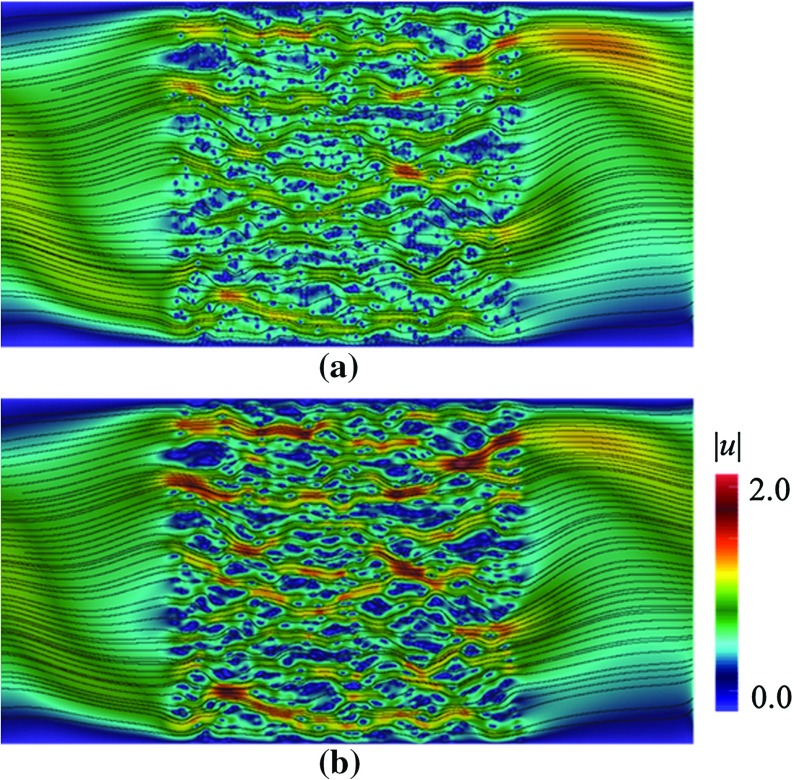
Contours of magnitude of velocity |*u*| along with their streamlines in a domain depicted in Fig. 3b, with $\mathbf {U}$ according to Eq. (18), computed using Crouzeix–Raviart MsFEM on **a** $128\times 64$ coarse elements; compared with **b** the reference solution

We consider a channel domain $\varOmega = [-2\le x \le 2, -1 \le y \le 1]$ containing a porous medium spanning from $x=-1$ to $x=1$. We then assign $\rho =1$, $\mu = 0.001$, and $\mathbf {f}=0$. At the inlet, the theoretical incompressible Poiseuille solution (parabolic velocity profile) is applied for all cases, i.e $\mathbf {u}=\left( 1-y^2,0\right)$ on $x=-2$, whereas the Neumann boundary condition $\partial u/\partial n = 0$ is assumed at the outlet, $x=2$. No–slip boundary conditions at the top and bottom walls are applied (Figs. 7, 8, 9).

**Fig. 10:**
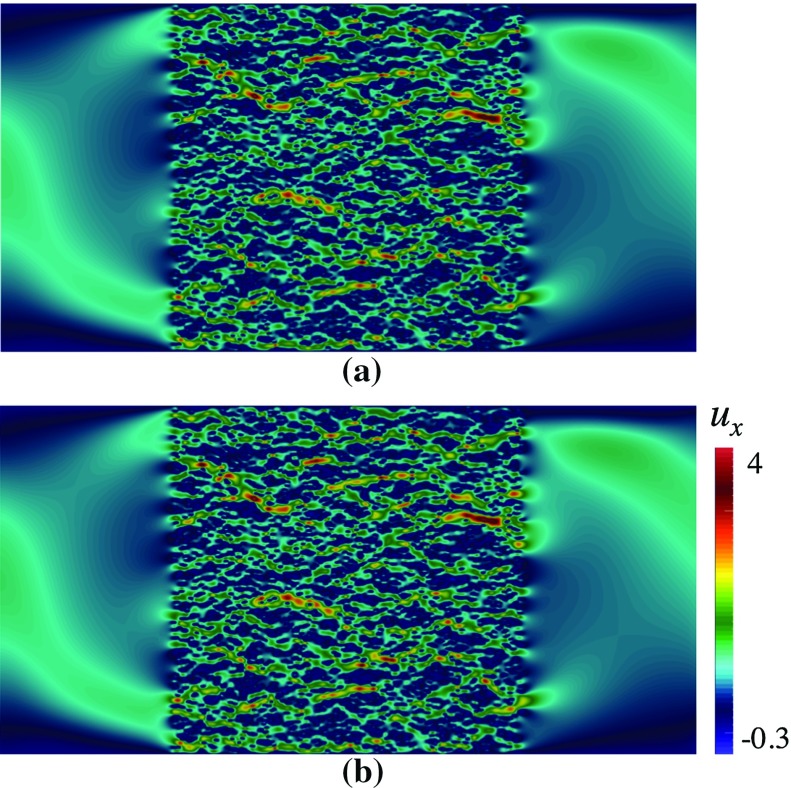
Contours of $u_x$ in a dense domain consisting of 3600 obstacles computed with **a** CR MsFEM on $256\times 128$; **b** reference solution on $2560\times 1280$. At 10 times coarser mesh, the result computed using CR MsFEM shows no noticable difference compared with the reference solution

First we apply our method on simple Poiseuille flow without any porous bodies. Then, two grain patterns are included as shown in see Fig. 3. From here on we refer to them as pattern (a) depicted in Fig. 3a, and pattern (b) depicted in Fig. 3b. Pattern (a) consists of 100 randomly placed grains, each with width $\varepsilon =0.06$. Pattern (b) consists of 900 grains with $\varepsilon =0.0067$ (Fig. 10).

We compare all our results to the reference solutions. When computing the reference solutions, we employ Q1-Q1 finite element method on a fine mesh consisting of $2560\times 1280$ quadrangles. This ensures the ratio $\varepsilon /h\ge 5$.

### Poiseuille Flow

**Fig. 11:**
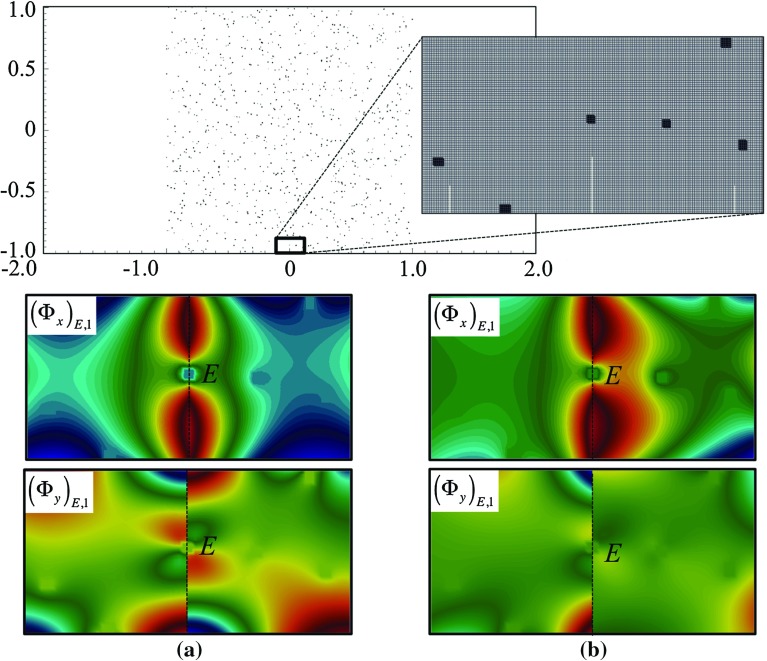
Multiscale basis functions $\mathbf {\Phi }_{E,j},j=1,2$ in the presence of grains of which, one coincides with the edge *E*. The basis functions depicted are for the given velocity field **a** $\mathbf {U}=0$; and **b** according to Eq. (18). In all cases, they successfully adapt to the worst-case scenario and maintain $\int _E \mathbf {\Phi }_{E,i}=\mathbf {e}_1$

We apply a vector field $\mathbf {U}=\left( 0.002,0\right)$ corresponding to ${Re}\approx 4$ where ${Re}=\frac{\rho \varepsilon |\mathbf {U}|}{\mu }$ (in the absence of grains, we assume that $\varepsilon$ is the channel diameter). In Table 1, the norms of error of the Crouzeix–Raviart MsFEM solutions relative to the reference solution on a number of coarse meshes are given, showing a convincingly converging trend (Fig. 11).

### Pattern (a)

Here, we test our method in solving flow pass a porous body with a random pattern of grains. We consider pattern (a) where each grain is a rectangle with width $\varepsilon = 0.06$, ${Re}\approx 0.12$. In Figs. 4 and 5, the contours of velocity components $u_x$ and $u_y$ computed on a number coarse meshes are given. The results are compared to the reference solution. The contours computed using Crouzeix–Raviart MsFEM at $256\times 128$ are identical to the reference solution; however, even at $32\times 16$, the flow features already resemble those of the reference solution, and at $64\times 32$ without any appreciable difference. Norms of error relative to the reference solution in $L^1, L^2$, and $H^1$ spaces are given in Table 2 showing a converging behaviour.

### Pattern (b)

Here, we simulate flow pass pattern (b) which contains finer ($\varepsilon = 0.0067$) and much more grains. We use the same kinds of coarse meshes, and vector field $\mathbf {U}$ as in the previous tests, which corresponds to ${Re}\approx 0.013$. This is a more challenging test than the previous ones due to much larger $H/\varepsilon$ ratios—ranging from 18.74 to 2.34. This means each coarse elements in the vicinity of the grains has higher chances of being occupied by more than one grain, and therefore suffers more oscillations.

In Figs. 6 and 7 the contours of $u_x$ and $u_y$ are given. As in the previous tests, the results are compared to the reference solution. The contours of ${u}_x$ and $u_y$ computed using Crouzeix–Raviart MsFEM at $256\times 128$ are obviously identical to those of the reference solution. The results at $64\times 32$ however already exhibit similar main flow features to the reference solution. This shows that the multiscale basis functions do well in capturing highly oscillatory fine-scale solutions. Similarly monotonically decreasing relative error norms are shown in Table 3. It is noted that the $H^1$ relative error, yielded using $32\times 16$ elements, may be large for this type of problem. If higher accuracy is needed at such a coarse setup, one can *enrich* the basis function spaces; for example, by using Generalised Multiscale Finite Element Method (GMsFEM; Efendiev et al. 2013).

**Table 1:** Convergence study of Poiseuille flow

$N_H$	$L^1$ relative	$L^2$ relative	$H^1$ relative
$32\times 16$	0.13	0.142	0.222
$64\times 32$	0.08	0.088	0.172
$128\times 64$	0.052	0.060	0.089
$256\times 128$	0.012	0.013	0.07

**Table 2:** Convergence study of channel flow pass pattern (a) with $\mathbf {U} = (0.002,0)$

$N_H$	$(H/\varepsilon )$	$L^1$ relative	$L^2$ relative	$H^1$ relative
$32\times 16$	2.08	0.144	0.170	0.433
$64\times 32$	1.04	0.097	0.121	0.347
$128\times 64$	0.52	0.054	0.071	0.282
$256\times 128$	0.26	0.011	0.024	0.173

**Table 3:** Convergence study of channel flow pass pattern (b) with $\mathbf {U} = (0.002,0)$

$N_H$	$(H/\varepsilon )$	$L^1$ relative	$L^2$ relative	$H^1$ relative
$32\times 16$	18.74	0.304	0.331	0.71
$64\times 32$	9.37	0.157	0.155	0.542
$128\times 64$	4.68	0.082	0.097	0.482
$256\times 128$	2.34	0.037	0.049	0.239

**Table 4:** Convergence study of channel flow pass pattern (b) with $\mathbf {U}$ according to Eq. (18)

$N_H$	$(H/\varepsilon )$	$L^1$ relative	$L^2$ relative	$H^1$ relative
$32\times 16$	18.74	0.32	0.355	0.80
$64\times 32$	9.37	0.153	0.161	0.567
$128\times 64$	4.68	0.077	0.102	0.442
$256\times 128$	2.34	0.038	0.043	0.319

### Setting with Dense Grains

Here, we simulate flow pass a porous body with dense grains. For the same grain size with that in pattern (b), $\varepsilon =0.0067$, we now lay 3600 grains randomly. The results computed with Crouzeix–Raviart MsFEM and the reference solution are given in Fig. 10. At 100 times coarser mesh, the result obtained using Crouzeix–Raviart MsFEM has no noticable difference compared with the reference solution.

### Heterogeneous Velocity Field *U*

To further test the feasibility of our method, we apply a heterogeneous velocity field

18 $$\begin{aligned} \mathbf {U}=\left( \begin{array}{rl} \begin{array}{cc} 2y(1-0.25 x^2)\\ -x(1-y^2) \end{array} \end{array}\right) ,\text { in } \varOmega _\mathrm{pore}. \end{aligned}$$

In Fig. 8, the contours of $u_x$ through pattern (b) computed using Crouzeix–Raviart MsFEM on $128\times 64$ coarse elements; and the reference solution are compared. The flow features are noticeably different than the previous results especially in regions away from the porous body. Indeed at the farthest from grains, the flow experiences ${Re}\approx 80$ where the inertial effect manifests the most. In Table 4, the convergence study is given for a range of coarse meshes. Again we can see that our method gives good qualitative and quantitative agreement with the reference solution. In Fig. 9, the contours of the magnitude of velocity computed using Crouzeix–Raviart MsFEM on $128\times 64$ coarse elements are displayed along with their streamlines and compared with the reference solution. We notice that the Crouzeix–Raviart MsFEM gives an excellent agreement in terms of flow pattern with the reference solution.

The effects of inertia away from the porous bodies can be instantly noticed when comparing the flow structures in Figs. 6 and 8 (computed on the same domain and grains structure, but with different $\mathbf {U}$). To help us understand the extent of these effects in a close vicinity of grains, we compare the fine-scale basis functions with, and without Oseen term. In Figs. 11, we plot the multiscale basis function $\mathbf {\Phi }$ computed in the framed, and zoomed patch that lies in a computational domain with pattern (b). Figure 11a shows the basis functions $\mathbf {\Phi }_{E,1}$ computed with $\mathbf {U}=0$—the Oseen problem therefore reduces to a Stokes problem. Figure 11b shows the basis functions $\mathbf {\Phi }_{E,1}$ computed with $\mathbf {U}$ according to Eq. (18). First, we note the effects of Oseen term on the shapes of the basis functions, which our method successfully captures. Second, in both cases the Crouzeix–Raviart basis functions succesfully accommodate the coincidence between a grain and an edge *E* and maintain $\int _E \mathbf {\Phi }_{E,i}=\mathbf {e}_i$.

## Concluding Remarks

The Crouzeix–Raviart MsFEM has been developed and tested for solving Oseen’s approximation for incompressible flow around solid bodies. The method performs very well in the presence of non-periodic grain formations. The weakly enforced continuity across coarse element edges ensures accurate basis function solutions without any oversampling methods. The basis functions are shown to successfully capture the effects of homogeneous and inhomogeneous vector field $\mathbf {U}$. The penalisation method is seamlessly incorporated into our method allowing an extensive utilisation of simple Cartesian mesh.

This method is developed as a stepping stone towards solving then more complicated Navier–Stokes equation. Although only two-dimensional cases are considered, the extension of this work on three-dimensions is straightforward. Similarly, the method can be applied for inhomogeneous Oseen’s problem with $\mathbf {f}\ne 0$. The reconstruction of fine-scale pressure is not the focus of the current work although it is possible (see Muljadi et al. 2015). The calculations of MsFEM basis functions within a coarse element are done independent of the neighbouring elements which makes it suitable for the application of parallel programming. The fine-scale discretisation of our method can be reformulated in a conservative fashion, which will improve accuracy. For example, following the proposition in Multiscale Finite Volume Method (MsFVM; Tomin and Lunati 2013; Jenny et al. 2003), or mass conservative Generalised Multiscale Finite Element Method (GMsFEM; Presho and Galvis 2016); this is one plausible future endeavour.

This work will lay the groundwork for applying various types of basis function *enrichment*. For example, *bubble* functions have been efficiently, and effectively integrated into the Crouzeix–Raviart MsFEM formulation (Degond et al. 2015) to solve advection–diffusion problems in a setting with very dense grains. A similar idea can be applied to the current method, and is the subject of our future work.

For practical applications, the method is a promising development towards simulations capable of handling a wide range of spatial scales, while accommodating nonlinear effects.
